# Benchmarking Point-of-Care Glucometers: A Comparative Study Using the Hexokinase Test and International Organization for Standardization (ISO) Standards

**DOI:** 10.7759/cureus.70981

**Published:** 2024-10-07

**Authors:** Kelita George, Sarah Joy, Haseena Kaladi Palliyalil, Shibin M Shaji, Vishakan Ajayakumar Sunanda, Aswathy Sreedevi

**Affiliations:** 1 Community Medicine, Amrita Institute of Medical Sciences, Amrita Vishwa Vidyapeetham, Kochi, IND; 2 Community Medicine, Amrita Institute of Medical sciences, Amrita Vishwa Vidyapeetham, Kochi, IND

**Keywords:** accuracy, blood glucose monitoring, diabetes management, glucometer, hexokinase

## Abstract

Introduction

The International Diabetes Federation states that India accounts for one in seven of all adults with diabetes. Adherence to self-monitoring of blood glucose is essential for effective management of diabetes. Despite the wide variety of glucometers and their clinical applicability, there is a lot of ambiguity regarding their accuracy. Therefore, this study aimed to evaluate if three point-of-care glucometers using three different methods for the estimation of blood glucose fulfil the minimum accuracy needed by ISO15197:2013 when compared with the gold standard hexokinase test.

Methods

A cross-sectional study was conducted at two primary health care centers in Ernakulam district, which included 73 participants with diabetes and 73 without diabetes. We evaluated three different enzymes in the test glucometers: glucose dehydrogenase with pyrroloquinoline quinone (GDH-PQQ), glucose dehydrogenase with flavin adenine dinucleotide (GDH-FAD), and glucose oxidase (GOD). The glucometer readings were compared with the gold standard hexokinase test and ISO15197:2013 standards. To compare the accuracy of each glucometer with the reference method, the Bland-Altman plot was used and to evaluate the clinical significance of the differences between the glucometer and reference value the Clarke error grid analysis was performed.

Results

When compared with the gold standard hexokinase result, only 78.08% (GOD), 92.4% (GDH-PQQ), and 95.8 (GDH-FAD) of results were within the limits stipulated by ISO15197:2013. All three glucometers showed blood glucose results within zones A and B of the consensus error grid, implying that it is clinically acceptable, and the Bland-Altman plot showed that the GDH-FAD method had the narrowest range between the upper and lower limit of agreement and a minimum bias of -0.89.

Conclusion

Only one test glucometer with the GDH-FAD method met the ISO15197:2013 criteria. Glucometers must meet the accuracy standards for the safety of the patients.

## Introduction

Diabetes currently affects an estimated 537 million adults worldwide between 20 and 79 years of age. By 2030, this number is projected to rise to 643 million and further increase to 783 million by 2045 [1]. In India, findings from the National Non-communicable Disease (NCD) Monitoring Survey reveal a diabetes prevalence rate of 9.3%, with 24.5% of the population exhibiting impaired fasting glucose [2]. Blood glucose measurement is central to diagnosing and managing diabetes, with self-monitoring of blood glucose (SMBG) at home playing a key role in helping people with diabetes improve glycemic control. The PRISMA (Prospective, Randomized Trial on Intensive Self-Monitoring Blood Glucose Management Added Value in Non-Insulin Treated Type 2 Diabetes Mellitus Patients) trial demonstrated that intensive SMBG significantly enhances glycemic control and aids in managing type 2 diabetes [3].

Monitoring of blood glucose by glucometer is convenient, cheap, and quick. The technology, usability, and accuracy of SMBG have advanced markedly since their introduction a few decades ago. Modern SMBG devices are compact and user-friendly, require only a small capillary blood sample, and deliver results within seconds [4]. It is critical that the glucometers measure glycemic levels with accuracy. This requires a reliable device that can measure glycemic levels with accuracy to detect both hypoglycemia and hyperglycamia. Many technological advances have been achieved in the recent past in enhancing these devices' accuracy, precision, portability, and memory. Yet, concerns continue to prevail regarding the accuracy of commonly used glucometers at the consumer level. Various glucometers use various types of mechanisms such as glucose oxidase (GOD) to measure blood sugar levels. GOD-based test strips were historically predominant, but studies indicate that these strips are significantly affected by oxygen levels [5].

To address these concerns, the International Organization for Standardization (ISO) has recommended specific guidelines for glucometer accuracy. However, many blood glucose monitors available in the market today fail to meet the current international standard, ISO 15197:2013 [6], or even its earlier, less stringent version, ISO 15197:2003. Despite the wide variety of point-of-care glucometers (PoCG) and their clinical applications, uncertainty and skepticism surround their use in clinical decision-making. Data on the accuracy of glucometers used in India, compared to the 2013 ISO standards, are limited. Additionally, there is a lack of studies from India comparing glucometers that use GOD and glucose dehydrogenase enzymes.

Hence, it is important to use glucometers that follow these standards. Since previous studies have shown differences in the accuracy with variability in the enzyme used in the glucometer, we have considered three glucometers in the Indian market, which use different enzymes and coenzymes. The objective of the study was to evaluate the accuracy of three PoCGs in comparison to the ISO 15197:2013 standards and gold standard hexokinase laboratory results.

## Materials and methods

A cross-sectional study was conducted among patients attending the OPD of two primary healthcare centers in Ernakulam district, Kerala. Individuals older than 18 years with diabetes and without diabetes were included in the study.

In the absence of previous studies, comparing glucometers in an Indian context, a pilot study was conducted among 20 participants (10 with diabetes and 10 without diabetes). We included three glucometers that use different glucose measurement technologies, specifically glucose dehydrogenase with pyrroloquinoline quinone (GDH-PQQ), glucose dehydrogenase with flavin adenine dinucleotide (GDH-FAD), and GOD methods, to assess their accuracy. The pilot study found that only 65% of the readings from GDH-PQQ, 90% from GDH-FAD, and 60% from GOD met the ISO 2013 standards. The current ISO 1597:2013 guidelines outline the performance criteria for glucometers when compared to results from a gold standard laboratory method. According to criterion 1, at least 95% of glucometer readings must be within ±15 mg/dL for glucose concentrations below 100 mg/dL and within ±15% for concentrations of 100 mg/dL or higher. As per criterion 2, in a consensus error grid analysis, at least 99% of glucometer results must fall within zones A and B [6]. All results of the three test glucometers during the pilot showed blood glucose levels within zones A and B of the Clarkes error grid. With 95% confidence, 15% relative error, and 10% non-response, a minimum sample size of 128 was calculated. We recruited 146 participants (73 with diabetes and 73 without diabetes) for the study. Each day alternate persons with and without diabetes were chosen for the study from each of the centers. Over a period of two months, enrolment of 73 patients with diabetes and 73 patients without diabetes was completed. Adults with a systolic BP higher than 100 mmHg (based on the patient’s history) were included in the study. Participants with a history of coagulopathy disorders, and hemolysed blood samples (according to laboratory reports) were excluded.

Sociodemographic information such as age, gender, and comorbidities were gathered using a structured questionnaire. Capillary blood samples were obtained via a single finger prick using a sterile lancet and were directly applied to the reagent strips of three different glucometers. Concurrently, intravenous blood samples were collected among all and were transported to the laboratory at the tertiary health care center by maintaining a cold chain. These samples were analyzed using the hexokinase method for blood glucose estimation at the main laboratory.

Statistical analysis

The data collected from the study were entered into Microsoft Excel (Microsoft Corp., Redmond, WA), and the analysis was conducted using SPSS Version 21 (IBM Corp., Armonk, NY). Descriptive statistics for continuous variables were summarized as mean and standard deviation. To compare the accuracy of each glucometer with the reference method, the Bland-Altman (B&A) plot was used. The B&A plot analysis is a simple way to evaluate a bias between the mean differences and to estimate an agreement interval, within which 95% of the differences of the second method when compared to the first method [7]. As for other relevant measures, it is recommended that 95% of the data points should lie within ±1.96 SD of the mean difference-limits of agreement. To evaluate the clinical significance of the differences between the glucometer and reference value, the Clarke error grid analysis was performed [8]. Error grid analysis is a method used to evaluate the clinical accuracy of glucometers. It categorizes readings into zones ranging from zone A to zone E. Readings in zone A indicate no impact on clinical decisions or treatment and are very safe for clinical use, while those in zone E suggest altered clinical actions that could lead to dangerous outcomes or incorrect treatment. The goal is for most glucometer readings to fall within zones A and B, indicating that the device is clinically accurate and safe for use.

Prior to the commencement of the study, clearance was obtained from the Institutional Ethics Committee ((ECASM-AIMS-2023-052). Privacy was ensured during the time of interview, and confidentiality of all the information collected was maintained. Participants provided written informed consent before data collection.

## Results

**Figure 1:**
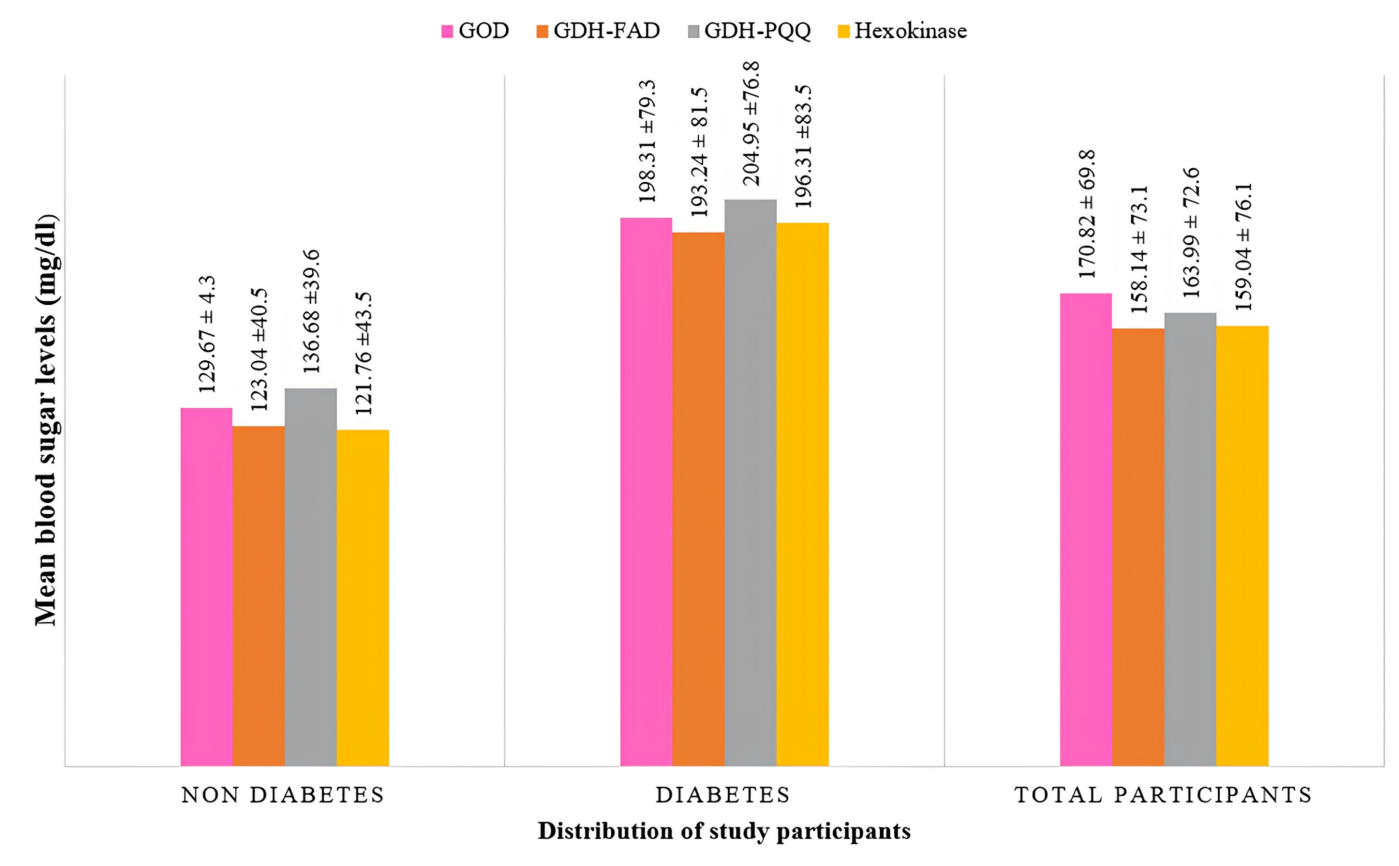
Distribution of study participants based on mean blood glucose values tested with the three test glucometers and the gold standard hexokinase test. GOD, glucose oxidase; GDH-FAD, glucose dehydrogenase with flavin adenine dinucleotide; GDH-PQQ, glucose dehydrogenase with pyrroloquinoline quinone

The study included 146 persons (73 with diabetes and 73 without diabetes), with a mean age of 60.11 ±12.63 years. More than half of the participants (56%) were females. The mean blood glucose levels for patients with diabetes and without diabetes were 196.31±83.51 mg/dL and 121.77±43.53 mg/dL, respectively, which was estimated using the hexokinase technique. The mean blood glucose values of the whole study population detected with the three glucometers, which used GDH-PQQ, GDH-FAD, and GOD, were 163.99±72.60 mg/dL,158.14±73.17 mg/dL, and 170.82 ±69.88 mg/dL, respectively. The mean blood glucose level of the 146 participants detected with the gold standard hexokinase was 159.04 ±76.18 mg/dL (Figure 1).

**Figure 2:**
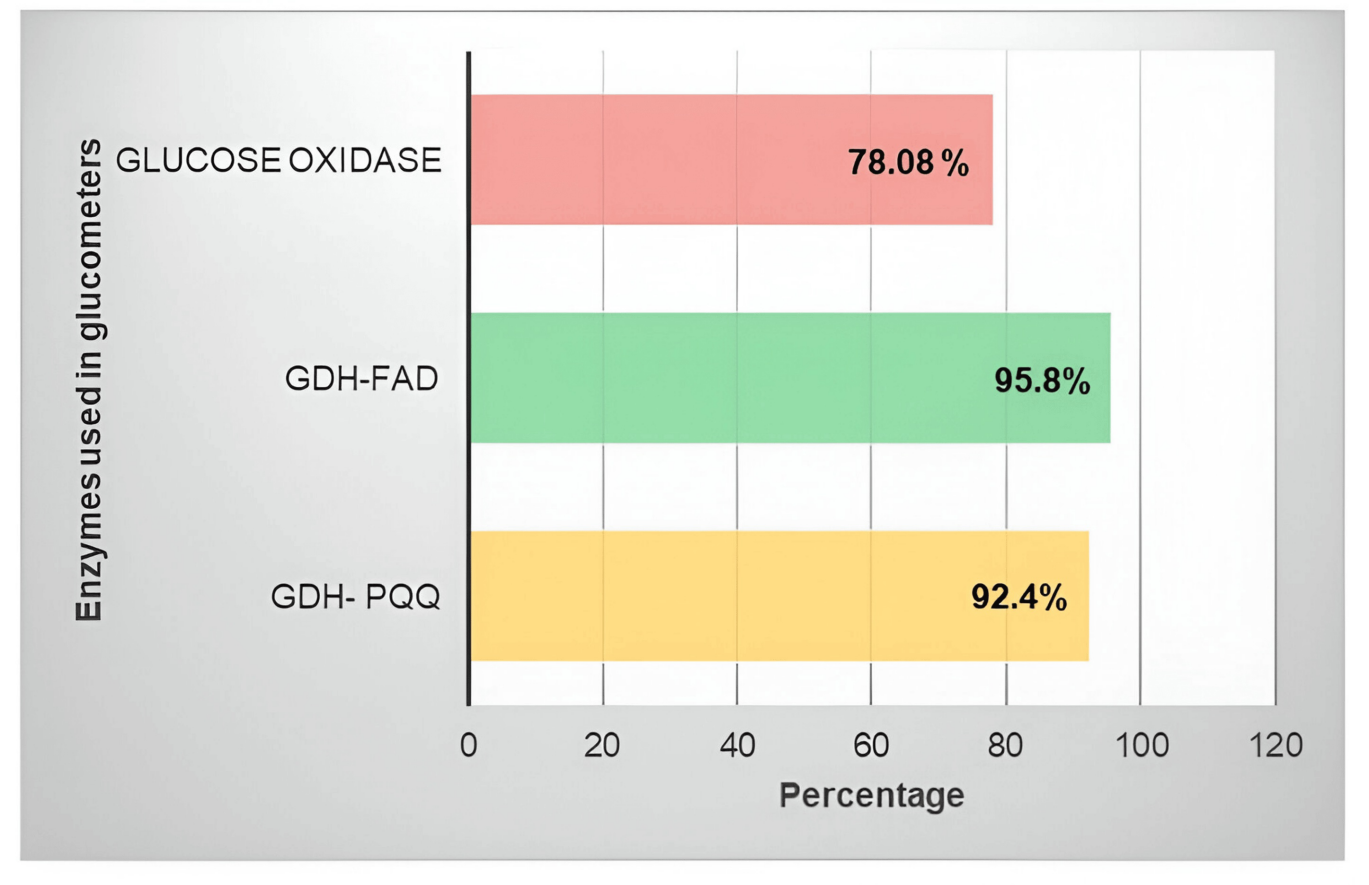
When tested with the test glucometers, only one glucometer that used the GDH-FAD method showed that more than 95% of results were within ISO standards GDH-FAD, glucose dehydrogenase with flavin adenine dinucleotide; GDH-PQQ, glucose dehydrogenase with pyrroloquinoline quinone

To meet ISO standards, glucometers must ensure that at least 95% of their readings fall within ±15 mg/dL for glucose levels below 100 mg/dL and within ±15% for levels of 100 mg/dL or higher. In our evaluation comparing glucose readings from each glucometer to the hexokinase method, only the GDH-FAD method glucometer achieved the required 95% compliance in accordance with the criteria stipulated by ISO15197:2013. In contrast, the GDH-PQQ and GOD method glucometers had compliance rates of 92.4% and 78.08%, respectively (Figure 2).

A B&A plot is a tool used to visualize the differences in measurements between two instruments or measurement techniques. It helps assess how closely a method aligns with the one currently in use or with the gold standard test. The plot’s X-axis represents the average or mean measurement of the two instruments, while the Y-axis shows the difference between their measurements. The plot also includes the average difference in measurements (referred to as the "bias"), and the upper and lower limits of the 95% confidence interval for this average difference. The "bias" line in the middle of the chart reflects the overall difference between the two instruments, with larger deviations from 0 indicating greater disparity in measurements. The upper and lower confidence interval lines give an idea of the typical range of agreement, where 95% of the differences generally fall within these limits. A wider confidence interval suggests a broader range of differences between the instruments.

**Figure 3:**
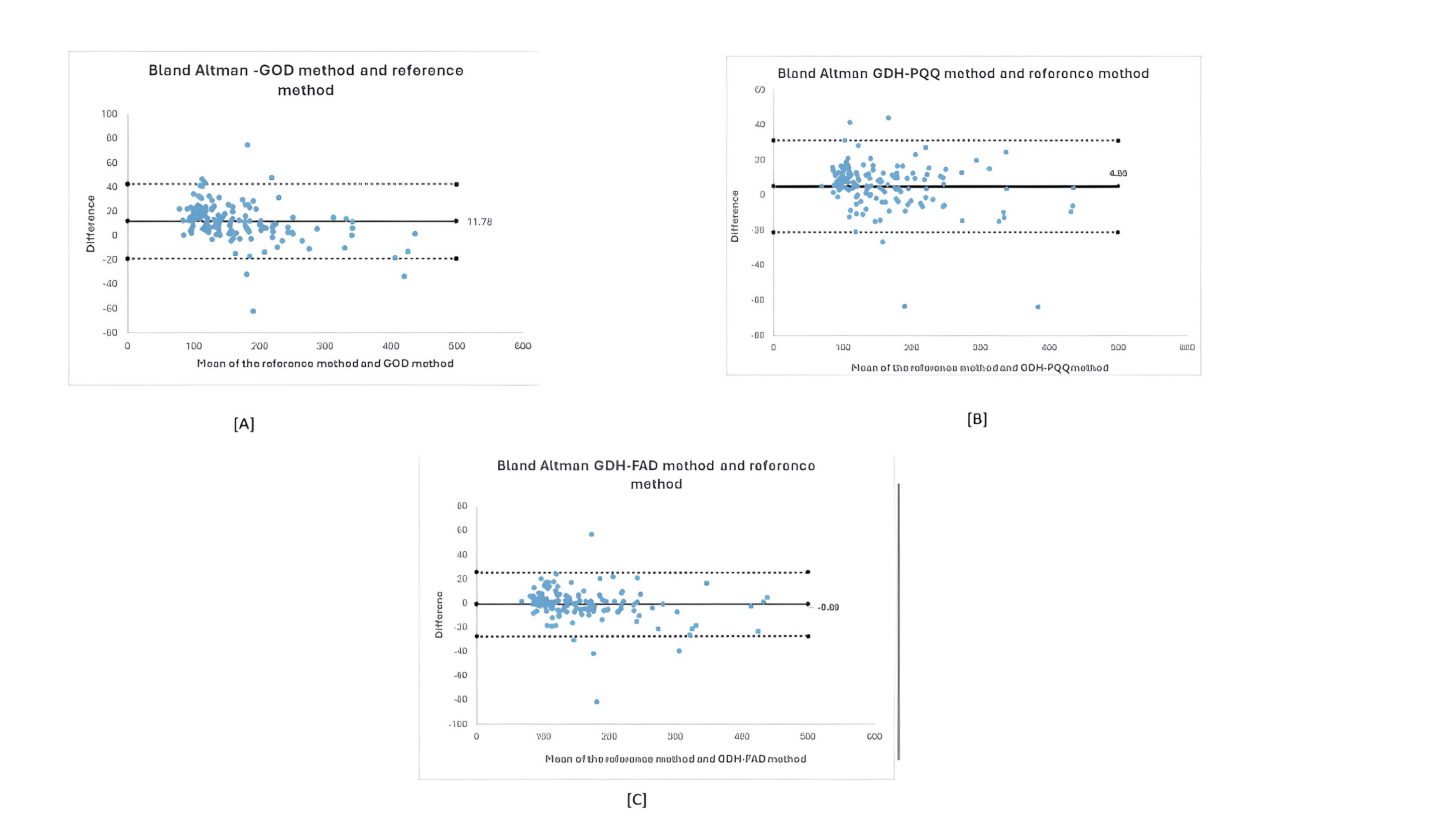
The Bland-Altman plot for the three glucometers. GOD, glucose oxidase; GDH-PQQ, glucose dehydrogenase with pyrroloquinoline quinone; GDH-FAD, glucose dehydrogenase with flavin adenine dinucleotide

Thus, the B&A plot used for this study compares the three test glucometer methods with the reference method: gold standard hexokinase method. GDH-PQQ method showed a bias of +4.95, GDH FAD method showed only a minimum bias of -0.89, and GOD showed a bias of +11.78 in the B&A plot. Thus, the GDH-PQQ and GOD methods overestimated the test values. The extent to which the glucometers deviated from the reference method is represented in B&A plots (Figures 3A-3C).

The difference of the test glucometer measurement and the reference hexokinase measurement is plotted. The X-axis represents the mean of the two measurements, and the Y-axis shows the difference of the two measurements. All values are in mg/dL. The dotted lines which represent upper and lower limits of agreement is calculated by mean difference ± 1.96 standard deviation of the differences. In a B&A plot, the upper and lower confidence interval lines give an idea of the typical range of agreement, where 95% of the differences generally fall within these limits. A narrow confidence interval suggests a more preferable lesser range of differences between the two methods, which was noticed in GDH-FAD.

In our study, the GDH-PQQ method showed a bias of +4.95, the GDH-FAD method showed only a minimum bias of -0.89, and GOD showed a bias of +11.78 in the B&A plot. Thus the GDH-PQQ and GOD methods overestimated the test values.

**Figure 4:**
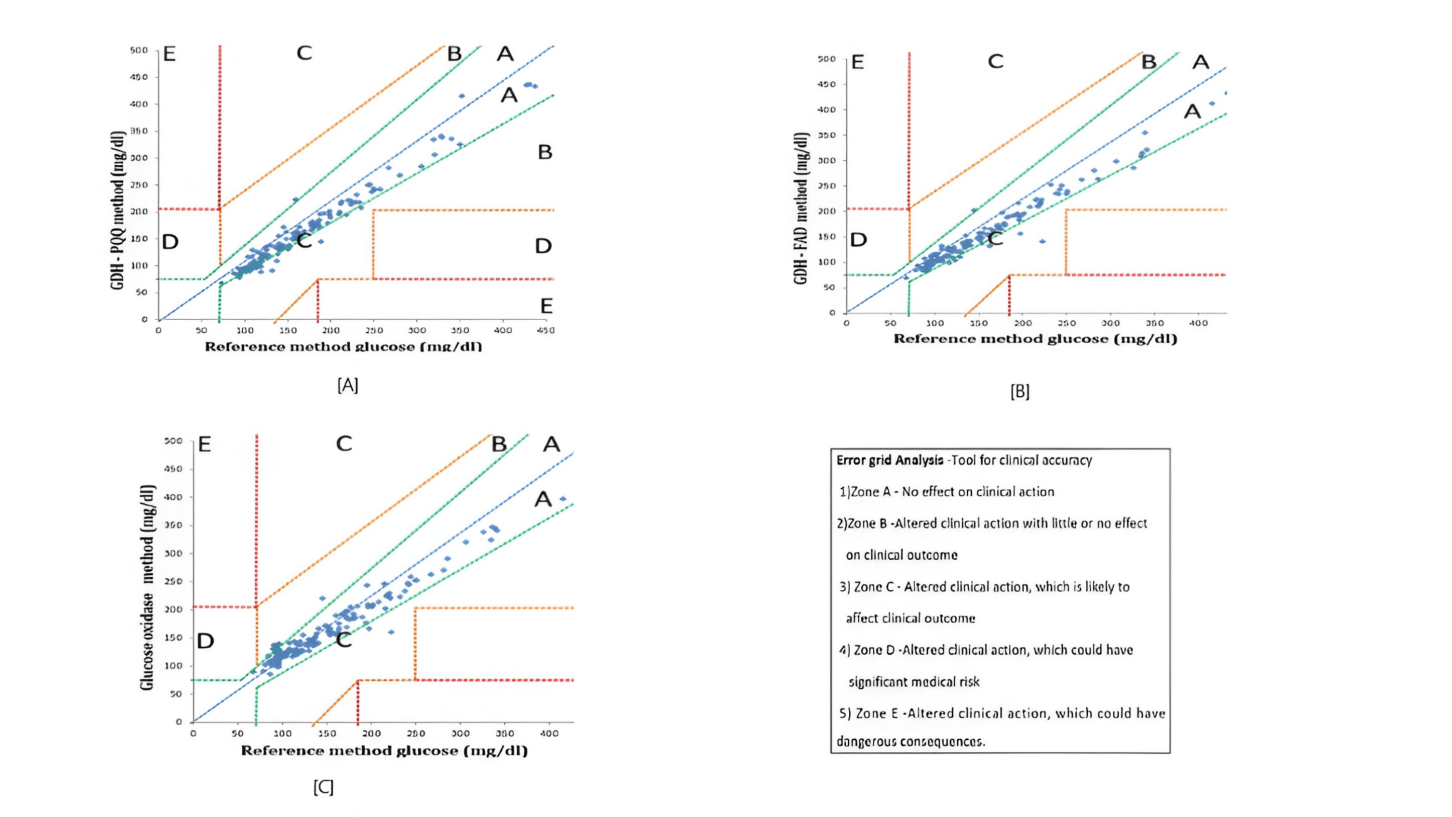
Clarke error grid analysis for clinically significant accuracy of glucometers. GDH-PQQ, glucose dehydrogenase with pyrroloquinoline quinone; GDH-FAD, glucose dehydrogenase with flavin adenine dinucleotide

The B&A plot method only defines the intervals of agreements; it does not say whether those limits are acceptable or not. Hence, for clinical accuracy, Clarke grid analysis was conducted. The Clarke error grid analysis indicated that all three glucometers had adequate clinical accuracy with all measurements in zones A and B, indicating no or limited effect on clinical outcome due to the variation noted from the gold standard test (Figures 4A-4C).

## Discussion

This observational study was conducted to test the accuracy of three glucometers with three different methods of estimation of blood glucose in comparison with the gold standard hexokinase test. The study conducted on 73 individuals with diabetes and 73 individuals without diabetes showed that only the glucometer that used GDH-FAD conformed to the ISO 2013 guidelines, although the error grid analysis of all three glucometers showed that values were within zones A and B. The B&A plot was used, which showed that that 95% of the data points were within ±1.96 SD of the mean difference-limits of agreement when compared to the reference method, and the maximum bias was observed for the GOD method and it overestimated the values on an average of around 11 mg/dL.

SMBG has surged in popularity among persons with diabetes. This can assist persons with diabetes to achieve optimum blood glucose control, particularly for those using intensive insulin regimens to minimize hypoglycemia and manage hyperglycemia [9]. Indian markets are flooded with several glucometers that use different methods and enzymes for estimating blood glucose values, and hence it is of utmost importance to know the accuracy of these methods and enzymes to choose an appropriate glucometer for optimal management of diabetes.

 A study conducted in Ethiopia showed that none of the four glucometer devices used for the study fulfilled the minimum accuracy measurement set by ISO 15197:2003 and ISO 15197:2013 standards. In addition, the linear regression analysis revealed that all four selected PoCGs overestimated the glucose concentrations [10]. A study conducted in Germany showed that out of 34 blood glucose systems assessed, only half fulfilled the minimal accuracy requirements of the ISO 15197 standards [11]. In a study conducted in Sri Lanka on eight commonly used glucometers, none of the glucometers fell within the ISO 2013 recommendations [12]. In another study conducted in Bangalore, India, in terms of accuracy, none of the nine glucometers tested satisfied the most stringent ADA-1994 standard, though all the glucometers showed improved accuracy with respect to the more relaxed ISO 1597:2003 standard [13].

In this study, the two GDH-based glucometers outperformed the GOD glucometer, and similar findings echo globally. In a study conducted by De Mol et al., this variation between the GDH and GOD glucometers was noticed only in higher altitude [14]. A review analysis in California found that among the entire cohort of tested glucometers, those who used a GDH test strip chemistry outperformed those who used GOD test strip chemistry for both ISO 15197 2003 and ISO 15197 2013 [15].

In a study comparing the two GDH-based glucometers conducted by Huang et al., the FAD-GDH-based monitor performed better than PQQ-GDH in neonates and patients with and without diabetes [16]. Thus similar findings were found in this study where the FAD-GDH glucometer outperformed proving the superiority of the same and proving that choosing a glucometer with the FAD-GDH method may benefit the user more than the other methods.

In our study, we evaluated both B&A and error grids for technical and clinical accuracy. While technical accuracy by the B&A plot defines meter performance, clinical accuracy tested by error grid establishes how treatment decisions agree between meter results and laboratory glucose results. Even though the technical accuracy of the two other glucometers that did not use GDH-FAD methods was not in power with the ISO standards, the clinical accuracy evaluation showed that all the values were within the safe zones of A and B. Most of the glucometer values were predominantly in zone A, indicating a deviation of 20% or less from the reference value. Very few glucometer values fell in zone B, signifying no or only benign change of treatment.

The limitation of this study is that blood glucose level is affected by many other factors such as patient status, examiner characteristics, and environmental conditions. However, these were taken care of to a great extent by carrying out the tests in similar environmental conditions and a skilled laboratory technician performed the tests. The findings are only applicable to the glucometers using these specific methods.

## Conclusions

The glucometer that used the GDH-FAD method outperformed the other two glucometers that used the GDH-PQQ and GOD methods by fulfilling the cut-offs of the criteria mentioned by ISO 2013. The technical accuracy was also found to be better in GDH-PQQ, while all three glucometers were found to be within safe limits in terms of clinical accuracy. Glucometers should be evaluated before use, and the specific meter model selected should be based on technical and clinical performance in the intended patient population for which many more studies that can evaluate the commonly available glucometers in the country are needed. This study highlights the importance of rigorous quality assurance and post-marketing surveillance in the country.

## Appendices

Sociodemographic Questionnaire

1) Name:

2) Age:

3) Gender:

4) Education:

5) Occupation:

6) Marital Status:

7) Location of house:

a. Rural      b. Urban

8) Color of ration card:

a. White     b. Blue     c. Pink    d. Yellow

9) Do you have diabetes:

a. Yes        b. No

10) If yes, duration of diabetes:

11) History of comorbidities:

a. Yes        b. No

12) If yes, specify:

13) Height:

14) Weight:

15) Body mass index:

16) Contact number:
